# Nephropathy 1st inhibits renal fibrosis by activating the PPARγ signaling pathway

**DOI:** 10.3389/fphar.2022.992421

**Published:** 2022-10-21

**Authors:** Linjie Mu, Liting Zhu, Yuan Feng, Nianzhao Chen, Feng Wang, Lijuan He, Jinguo Cheng

**Affiliations:** ^1^ Zhejiang Chinese Medical University Affiliated Wenzhou Hospital of Traditional Chinese Medicine, Wenzhou, Zhejiang, China; ^2^ The First Affiliated Hospital of Wenzhou Medical University, Wenzhou, Zhejiang, China; ^3^ Suzhou Wujiang District Hospital of Traditional Chinese Medicine (Suzhou Wujiang District Second People’s Hospital), Suzhou, China; ^4^ Xi’an TCM Hospital of Encephalopathy, Xi’an, Shanxi, China

**Keywords:** nephropathy 1st, renal fibrosis, PPARγ/klotho pathway, GW9662, kidney

## Abstract

Renal fibrosis is a manifestation of kidney injury. Nephropathy 1st is a traditional Chinese herbal medicine that has been used as a therapy for kidney disease, but the underlying mechanisms remain elusive. The aim of this study was to investigate the role and underlying mechanisms of Nephropathy 1st on the progression of kidney disease. In the present study, unilateral ureteral obstruction was performed to establish the renal fibrosis rat model. By hematoxylin–eosin staining and immunohistochemical staining analysis, the severity of renal fibrosis was evaluated *in vivo*. Serum creatinine (CREA) and urea nitrogen (BUN) were measured by ELISA. The expression levels of Col-I, FN, PPARγ, and Klotho were measured by Western blot in rat NRK-49F cells and in fibrotic rats. GW9662 was used to inhibit PPARγ signaling. Metabonomic analysis showed metabolic differences among groups. Nephropathy 1st administration alleviated the progression of rat renal fibrosis and reduced serum creatinine (Scr) and BUN levels. Mechanistically, Nephropathy 1st promoted the expression of PPARγ and thus activated PPARγ signaling, thereby reducing the pro-fibrotic phenotypes of fibroblasts. The therapeutic effect of Nephropathy 1st was abrogated by the PPARγ inhibitor GW9662. Moreover, Nephropathy 1st normalized the dysregulated lipid metabolism in renal fibrosis rats. In conclusion, Nephropathy 1st alleviates renal fibrosis development in a PPARγ-dependent manner.

## Introduction

Renal fibrosis is a pathological process characterized by the extraordinary accumulation of extracellular matrix in response to kidney injury. It has been recognized as the major contributor to various kidney diseases (Campanholle et al., 2013). Renal fibrosis is the common outcome of many chronic kidney diseases (CKD) independent of the underlying etiology (Nastase et al., 2018). To date, the underlying mechanisms of renal fibrosis remain controversial.

Despite numerous preclinical and clinical studies, currently available strategies only ameliorate or delay the progression of CKD but fail to reverse fibrosis. It is reported that myofibroblasts may arise from a number of sources such as activated renal fibroblasts (Li R. et al., 2020), pericytes (Yuan et al., 2019), epithelial-to-mesenchymal transition (EMT) (Su et al., 2020), endothelial-to-mesenchymal transition (EndoMT) (Sun et al., 2016), bone marrow-derived cells, and fibrocytes (Sun et al., 2016; Kuppe et al., 2021). Fibroblasts maintain a myofibroblast phenotype by expressing α-SMA and producing a large number of ECM components. Among various mediators, transforming growth factor-β (TGF-β) plays a critical role in tissue fibrosis by diverse mechanisms, such as activating downstream Smad2/3/7 proteins to regulate renal fibrosis through the classical pathway or through the non-classical pathway, regulating the activity of PI3K/RhoA/TAK1/Ras and other proteins to mediate renal fibrosis. In addition, transforming growth factor-β (TGF-β) can regulate the transformation of macrophages into myoblasts, a novel pathway affecting tissue fibrosis, by regulating these related pathways, upregulating matrix protein synthesis, inhibiting matrix degradation, and altering the cell–cell interaction (Gu et al., 2020).

Traditional Chinese herbal medicines have been reported to be useful in clinical kidney fibrosis treatment. For example, astragaloside IV was reported to attenuate podocyte apoptosis by inhibiting oxidative stress *via* activating the PPARγ-Klotho-FoxO1 signaling pathway, thereby ameliorating diabetic nephropathy (Xing et al., 2021). Fuzheng Huayu recipe, a traditional Chinese compound herbal medicine, significantly decreased kidney collagen deposition, hydroxyproline content, and type I collagen level *via* modulating miR-21/PTEN/AKT signaling (Wang et al., 2020). Shenkang VII recipe regulated the synthesis and degradation of the extracellular matrix and reduced the functions of pro-fibrotic fibroblasts by blocking the TGF-β1/Smad signaling pathways in UUO rats. As a herbal decoction, Nephropathy 1st is a compound preparation of Chinese herbal medicine, including Radix Bupleuri (10 g), *Scutellaria baicalensis* (12 g), *Pinellia ternata* (12 g), White Peony Root (30 g), glabrous greenbrier rhizome (30 g), *Scutellaria barbata* (30 g), Chinaroot Greenbier Rhizome (30 g), and Uniflower Swisscentaury Root (30 g), for the treatment of chronic nephritis independently developed by our members of the research group. Based on our previous studies on the mechanisms of Nephropathy 1st in renal fibrosis, we found that Nephropathy 1st effectively alleviates the progression of renal fibrosis through downregulating TGF- β1 expression and reducing the levels of Wnt4 and β-catenin. Additionally, Nephropathy 1st has achieved good curative effects in clinical use for Chinese populations. However, much remains unclear about the specific mechanisms of Nephropathy 1st in renal fibrosis development regulation. In this research, we aimed to explore the effects and mechanisms of Nephropathy 1st in renal fibrosis, in order to provide evidence for the better clinical application of this Chinese medicine.

## Materials and methods

### Sample collection of patients

A total of 10 patients with renal fibrosis were enrolled from The First Affiliated Hospital of Wenzhou Medical College, China. Percutaneous renal biopsy samples were obtained from 10 patients after a renal fibrosis diagnosis was confirmed, and renal biopsies from 10 patients without fibrotic lesions were used as controls. Patients were excluded if any of the following was present: polycystic kidney disease, pregnancy, human immunodeficiency virus, renal cancer, or recent immunosuppressive therapy. All patients had signed the informed consent forms. We collected the peripheral blood from each of the patients before and after taking Nephropathy 1st and further separated the serum to detect the creatinine (CREA) and urine protein by Abbott Aeroset (Abbott, Abbott Park, IL).

### Cell culture

The NRK-49F cell line was preserved by our laboratory. NRK-49F was cultured in Dulbecco’s modified Eagle’s medium with calf bovine serum to a final concentration of 5%. The cells were kept at 37°C with 5% CO_2_.

NRK-49F cells (Bioresource Collection and Research Center, Hsinchu, Taiwan) were plated in a 12-well plate at 5 × 105 per well. After 24 h, the complete medium was replaced by DMEM medium without bovine serum and cultured for another 24 h A measure of 500 μl of DMEM was left before adding 500 μl of the mixture containing TGF-β1 (with a final concentration of 10 ng/ml), FBS (with a final concentration of 5%), and a different dose of Nephropathy 1st (5, 15 and 25 µM) with three replicates. Cells were cultured for 72 h with Nephropathy 1st.

### Ureteral obstruction model and animal groups

A unilateral ureteral obstruction (UUO) rat model was performed in this study. In short, all rats were anesthetized with pentobarbital sodium (CAS:57-33-0; SIGMA-P3761) *via* intraperitoneal injection. Under anesthesia, an oblique incision was made from the back 1.5 cm away from the left costal angle. After incision, the left kidney was exposed and the left ureter was set free, double ligated at about the renal pelvis and 1/3 above the ureter, and the kidney was retracted and sutured. Rats in sham groups were treated as UUO without ligation after laparotomy. Male SD rats aged 6–8 weeks and weighing 185 ± 10 g were used for the experiments. All animal care and experimental procedures were reviewed and approved by the Animal Experiment Ethics Committee of Wenzhou Medical University. Rats were randomly divided into eight groups (n = 6 per group). Nephropathy 1st is composed of eight traditional Chinese medicines, including Radix Bupleuri (10 g), *Scutellaria baicalensis* (12 g), *Pinellia ternata* (12 g), White Peony Root (30 g), glabrous greenbrier rhizome (30 g), *Scutellaria barbata* (30 g), Chinaroot Greenbier Rhizome (30 g), and Uniflower Swisscentaury Root (30 g). These herbal drugs were searched in the TCMSP database (https://tcmsp-e.com/index.php) for their active ingredients and were screened by the relevant parameters oral bioavailability (OB) and drug-likeness (DL) with a threshold of OB ≥ 30% and DL ≥ 0.18. (0.18, the final active ingredients of the drugs are shown in Supplementary Table S1). Nephropathy 1st was diluted with purified water to the designated concentration of 1 mg/ml, and each rat was administered a volume of high concentration-18 mg/kg, median concentration-9 mg/kg, or low concentration-4.5 mg/kg. Group1 (sham): sham-operated rats were treated with saline *via* intragastric administration (i.g.) for 3 days and were set as control. Group 2 (UUO): the UUO group was treated with saline *via* i. p. for 3 days. Group 3 (UUO + Lotensin): UUO rats were treated *via* intragastric administration (i.g.) with lotensin (NOVARTIS, China) 1.67 mg/kg once a day for 2 weeks. Group 4 (UUO + Nephropathy 1st decoction-L): UUO rats were treated with a low dose of Nephropathy 1st decoction *via* i. g. once a day for 2 weeks. Group 5 (UUO + Nephropathy 1st decoction-M): UUO rats were treated with a medium dose of Nephropathy 1st decoction *via* i. g. daily for 2 weeks. Group 6 (UUO + Nephropathy 1st decoction-H): UUO rats were treated with a high dose of Nephropathy 1st decoction *via* i. g. daily for 2 weeks. Group 7 (UUO + GW9662–0.5 mg/kg): UUO rats were treated with the PPARγ inhibitor GW9662 (CSNpharm, USA) *via* i. g. once a day for 2 weeks. Group 8 (UUO + GW9662 + Nephropathy 1st decoction-M): UUO rats were treated with the PPARγ inhibitor GW9662 for 30 min before a medium dose of Nephropathy 1st decoction treatment for 2 weeks. Two weeks after UUO, mice were sacrificed. A portion of the left kidney (renal tissue) was fixed in 10% neutral-buffered formalin and 2.5% glutaraldehyde phosphate buffer (pH 7.4) for pathology, immunohistochemical (IHC) analyses, and morphological examination.

### Hematoxylin–eosin staining analysis

Hematoxylin–eosin staining was conducted according to protocols as mentioned (Guo et al., 2016). After deparaffinization and rehydration, sections were stained with hematoxylin dye solution for 5 min and rinsed with tap water for 3 min, followed by differentiation with 1% hydrochloric acid alcohol for 5 s and rinsing with tap water for 3 min. Then, it was followed by dyeing with 1% eosin alcohol for 1 min and washing with distilled water for 2 min. The sections were then dehydrated with graded alcohol and cleaned in xylene. The slides were then photographed by using a Nikon fluorescence microscope (Tokyo, Japan).

### Sirius red staining

According to the manufacturer’s instructions, Sirius Red staining was performed using the Picro-Sirius Red Stain Kit (Abcam, United States). Samples were processed by the Department of Anatomical Pathology, The First Affiliated Hospital of Wenzhou Medical College. ImageJ software was used to measure the percentage of the entire cortex that was occupied by collagen in Picro-Sirius Red-stained sections.

### Masson staining

Deparaffinized and rehydrated paraffin-embedded renal sections were stained with Masson staining for the analysis of renal fibrosis. In order to evaluate collagen deposition, the sections were stained with 0.1% Masson staining buffer. Images were taken digitally by ImageJ software (Media Cybernetic, USA) to determine the respective Masson-stained (fibrosis) and non-Masson-stained (normal) areas of the sections.

### ELISA assay

Using the ELISA commercial kits (The Seno Clinical Diagnostic Products Co., Japan), after 14 days of administration, the rats were placed in a clean metabolic cage to collect 24 h urine. The proteins in urine were measured by the biuret method. Serum creatinine (CREA) and urea nitrogen (BUN) concentrations were measured as well.

### Immunohistochemistry

The TGF-β1 expression level in the rat kidney was measured by immunohistochemistry analysis. The procedures were carried out according to the instructions of the Dako REAL EnVision Detection System (K5007, Dako, Denmark). The TGF-β1 expression level was quantified by ImageJ software.

### Western blotting

Cells from rat kidneys or the NRK-49F cell line were lysed by RIPA lysis buffer on ice for 15 min and then centrifuged at 12,000 rpm for 15 min at 4°C. The pellets were separated from the supernatants and mixed with 5× loading buffer. The mixture was isolated by SDS-PAGE, followed by transfer to PVDF membranes. The membranes were washed by 0.5% TBST twice, followed by incubation with 5% nofat dry milk for 2 h at room temperature. Membranes were washed by 0.5% TBST twice before being incubated with anti-PPARγ (1:1,000, Affinity, USA), anti-klotho (1:1,000, Affinity, United States), anti-TGF-β (1:1,000, Abcam, United States), anti-COI-I (1:1,000, Abcam, United States), anti-FN (1:1,000, Abcam, United States), anti-α-SMA (1:1,000, Affinity, United States), and anti-GAPDH (1:5,000, China) overnight at 4°C. On the other day, the membranes were washed by 0.5% TBST three times and then incubated with HRP-ligated goat anti-rabbit second antibodies (1:5,000, Boster, China) for 2 h at room temperature. The blots were detected by an enhanced chemiluminescence method. ImageJ software was used for band densitometry. GAPDH expression in each sample was set as a loading control.

### RNA-seq and genome-wide transcriptome analysis

Total sample RNA from renal tissues was isolated and purified using TRIzol (Thermofisher, 15,596,018), according to the manufacturer’s protocol. Total RNA was then quality controlled for quantity and purity with a NanoDrop ND-1000 spectrophotometer (NanoDrop, Wilmington, DE, USA) and checked for integrity with a Bioanalyzer 2100 system (Agilent, CA, USA); concentrations >50 ng/μL, RIN value > 7.0, and total RNA>1 μg are sufficient for downstream experiments. PolyA-bearing mRNA was specifically captured by two rounds of purification using oligo(dT) magnetic beads (Dynabeads Oligo(dT), cat. 25–61005, Thermo Fisher, United States). The captured mRNA was fragmented using the Magnesium Ion Fragmentation Kit (NEBNext^®^ Magnesium RNA Fragmentation Module, cat. E6150S, USA) at a high temperature of 94°C for 5–7 min. The fragmented RNA was synthesized into cDNA by reverse transcriptase (Invitrogen SuperScript™ II Reverse Transcriptase, cat. 1,896,649, CA, United States). Then, double-strand synthesis was performed using *E. coli* DNA polymerase I (NEB, cat. m0209, United States) and RNase H (NEB, cat. m0297, United States), and these complex duplexes of DNA and RNA were converted into DNA duplexes, while dUTP solution (Thermo Fisher, cat. R0133, CA, United States) was incorporated into the double-stranded DNA to blunt the ends of the double-stranded DNA. An A base is added at each end to allow it to be connected to a linker with a T base at the end, and the size of the fragments is screened and purified by magnetic beads. The second strand was digested with UDG enzyme (NEB, cat. m0280, MA, US) and then by PCR—pre-denaturation at 95 °C for 3 min, denaturation at 98 °C for a total of eight cycles of 15 s each, annealing to 60 °C for 15 s, extending at 72 °C for 30 s, and finally extension at 72 °C for 5 min to form a library with a fragment size of 300bp ± 50bp (strand-specific library). Finally, we pair-end sequenced it using the Illumina Novaseq™ 6000 system in the PE150 sequencing mode using standard procedures.

### Data preprocessing and differentially expressed gene (DEG) screening

The limma R package (version 3.36.5) of Bioconductor 3 (https://www.bioconductor. org/pack-ages/release/bioc/html/limma.html) was adopted to conduct the quantile normalization of the raw data and subsequent data processing to identify the DEGs between the UUO and the controls. The DEGs between the two groups were evaluated using t-tests, and the *p*-values were corrected for the false discovery rate (FDR) by using the Benjamini–Hochberg (BH) procedure. Only genes with a |log2fold change (FC)|>1 and FDR <0.05 were selected. Volcano plot filtering was applied to visualize the significant DEGs. The differential gene expression patterns between the two sample groups were analyzed by hierarchical clustering.

### Functional and pathway enrichment analysis of the DEGs

clusterProfiler V3.8 is a bioconductor-dependent R software package that not only automates the biological term classification process and gene cluster enrichment analysis but also provides a visualization module that displays the results of the analysis. In the present study, the clusterProfiler package was used for Gene Ontology (GO) and Kyoto Encyclopedia of Genes and Genomes (KEGG) enrichment analyses of the identified DEGs.

### Metabolic analysis

Thermo Scientific UltiMate 3000 HPLC and UHPLC systems, Bruker Impact II UHR-QqTOF (ultra-high resolution Qq-time-of-flight) mass spectrometry, were used for metabolic analysis in the rat serum. XCMS was used to extract the spectral peak area, and MetaboAnalystR 2.0 was used for spectral peak analysis. The xcms package and the centwave algorithm were used to identify spectral peaks (PPM = 5). After correcting RT, peak matching was conducted for 30 samples again. The missing value adopts the fillChromPeaks method, and the intensity value of such features in the missing samples is defined by the integral signal in the MZ RT region of the feature. Finally, 2,493 peaks (MZ/RT) are identified. The MetaboAnalystR package was used to replace the value of missing or zero peak intensity with half of the minimum positive values in the original data. Denoising: the IQR algorithm was used to filter out 25% of the features with constant expression in all samples.

We screened possible marker peaks using the following conditions: (group 1/group 2) cross ((group 2/group 4) and (group 2/group 5) and (group 2/group 6)) cross ((non (group 1/group 4)) and (non (group 1/group 5)) and ((group 1/group 6))). Here, (group 1/group 2) represents the significant change spectrum peak obtained by comparing group 1 with group 2. We used the *t*-test and the PLS-DA method to evaluate the significance of the change. The threshold is set to FDR <0.05 and VIP >1. Intersection and union are set operations.

### Immunofluorescence

For immunolocalization, cells were fixed in 4% paraformaldehyde, followed by permeabilization with 0.2% Triton X-100. After blocking using normal goat serum, primary antibody against α-SMA (1:1,000; ab7817, Abcam, United States) and collagen III (1:2000; ab7778, Abcam, United States) was incubated with the cells. Then, the cells were incubated with the corresponding Alexa Fluor-conjugated secondary antibody for 1 h. Images were taken using a fluorescent microscope.

### Statistical analysis

Statistical analysis was performed by SPSS 21.0 (SPSS, Inc., USA) and GraphPad Prism 9.0 (GraphPad Software, USA). The data were expressed as the mean ± SD. Each experiment was repeated at least three times, and the representative experiment was shown. The statistical significance of differences was calculated using the one-way analysis of variance (ANOVA), and *p* < 0.05 was considered statistically significant.

## Results

### Nephropathy 1st attenuated rat renal fibrosis *in vivo*

**FIGURE 1:**
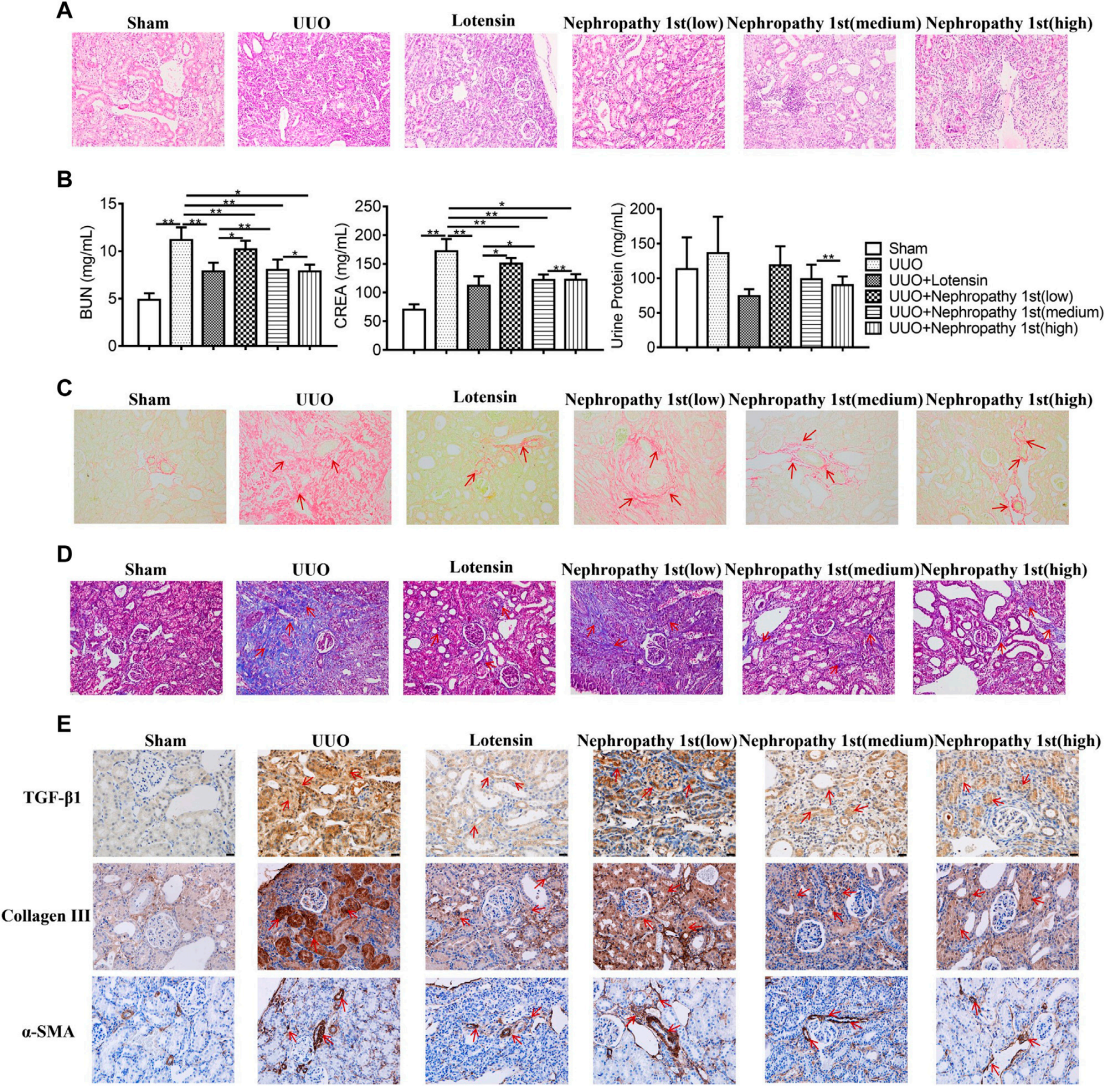
Nephropathy 1st inhibited TGF-β1-induced myofibroblast formation. **(A)** HE staining of rat kidneys. **(B)** Serum creatinine (CREN), urea nitrogen (BUN), and urine protein concentrations were measured in the blood of rats. **(C)** Sirius Red staining and **(D)** Masson staining of different kidney tissues. **(E)** IHC staining of TGF-β1, collagen III, and α-SMA in rat kidneys. Data were representative of three independent experiments. Data were shown as mean ± SD, *p < 0.05 and **p < 0.01.

To elucidate the role of Nephropathy 1st in renal fibrosis, we established the rat UUO model. Then, the rats were treated with the low, medium, and high concentrations of Nephropathy 1st and lotensin in the therapeutic groups. HE staining and renal injury score were used to evaluate the kidney pathology. As shown in Figure 1, rats in the UUO model group have the highest renal injury score. The morphology of renal tubules and glomeruli was unclear, the interstitium was thickened, and massive inflammatory cell infiltration was observed in the kidney, suggesting that renal fibrosis was successfully induced. Lotensin, a widely used anti-renal fibrosis drug (Geng et al., 2018; Dong et al., 2019; Zhang et al., 2019), significantly inhibited the development of renal injury. Notably, Nephropathy 1st administration relieved the severity of renal injury in a dose-dependent manner. Compared with the sham group, rats from the UUO group exhibited higher levels of serum creatinine (CREA) and urea nitrogen (BUN). No difference in urine proteins was observed among groups. As a positive control, lotensin suppressed CREA and BUN levels in the serum. Similar to lotensin, the medium and high concentration of Nephropathy 1st also reduced the serum levels of CREA and BUN (Figure 1B).

Next, Sirius Red staining was performed to detect the collagen fiber. Sirius Red positive staining was notably found in renal tissues in the model group, which was reduced by lotensin treatment. All the three concentrations of nephropathy 1st inhibited collagen fiber formation, and the therapeutic effect of the medium and high concentrations of nephropathy 1st was similar to that in the lotensin group (Figure 1C). Masson staining further indicated that lotensin could reverse renal fibrosis induced by UUO treatment. Nephropathy 1st also showed a similar effect, especially at medium or high concentrations (Figure 1D). TGF-β1, collagen III and α-SMA play critical roles in the progression of renal fibrosis. The IHC results revealed that TGF-β, collagen III, and α-SMA were all highly expressed in the renal tissues after UUO treatment. As expected, lotensin remarkably inhibited these changes. Again, Nephropathy 1st dose-dependently inhibited the expression of TGF-β1, collagen III, and α-SMA (Figure 1E). Importantly, the serum levels of creatinine and urea nitrogen were significantly decreased after nephropathy 1st treatment (Figure 1B). Hence, Nephropathy 1st inhibited TGF-β1-induced myofibroblast formation.

### Nephropathy 1st restored PPARγ expression in the fibrotic kidney

**FIGURE 2:**
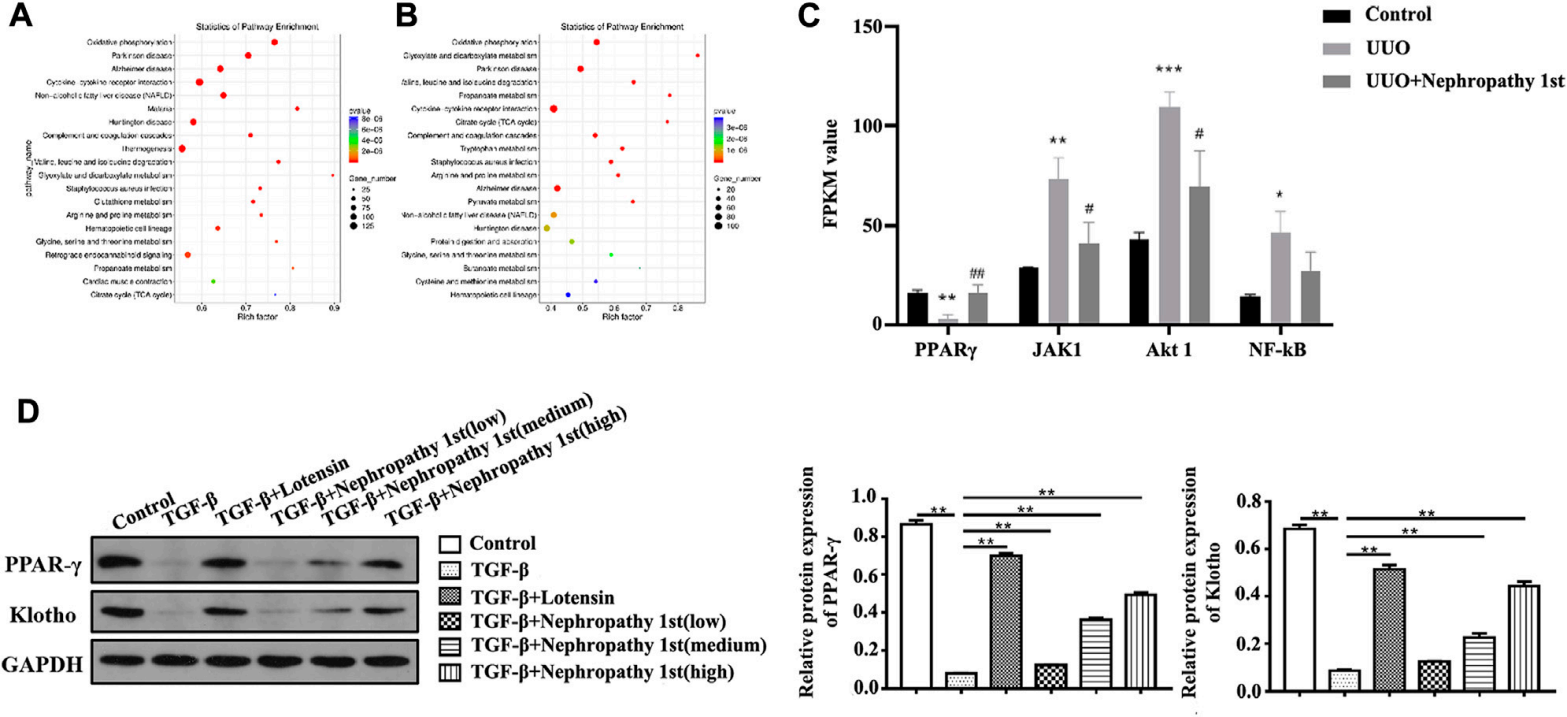
RNA-seq analyze the effect of Nephropathy 1st on PPARγ expression. The scatter diagram shows the KEGG enrichment analysis of different genes between control vs. UUO **(A)** and the UUO vs. Nephropathy 1st **(B)** group. The expression of different genes from the pathway of oxidative stress inflammation using NGS. **(C)**. Expression of PPARγ, JAK1, Akt1, and NF-κB in different groups was detected. **(D)**. Western blot was performed to assess the expression of PPARγ and Klotho. Data were representative of three independent experiments. **p < 0.01.

Next, we wondered the molecular mechanisms through which Nephropathy 1st exerts its anti-fibrosis role. RNA-seq was carried out to explore the mRNA expression profile by high-throughput sequencing (NGS). According to the differentially expressed genes (DEGs), we performed KEGG pathway enrichment analyses (Figures 2A–C) and found that the DEGs were mainly enriched in the pathways related to lipid metabolism, such as oxidative phosphorylation and lipid peroxidation. PPARγ is the central transcription factor controlling lipid metabolism. We found that the PPARy renal expression was significantly lower in the UUO model compared with the control group and treatment with Nephropathy 1st, and the standard drug restored PPARγ expression. Moreover, the expression of JAK1, AKT1, and NF-κB was significantly increased in the model group, and there was low expression in the Nephropathy 1st group. Furthermore, Western blot was performed to assess the expression of PPARγ. In fibroblasts, TGF-β1 treatment significantly inhibited the expression of PPARγ and klotho—a downstream target of PPARγ. Lotensin and the medium and high concentrations of Nephropathy 1st increased the levels of PPARγ and klotho in TGF-β1-challenged fibroblasts (Figure 2D), suggesting that PPARγ might be involved in the therapeutic effect of Nephropathy 1st.

### Nephropathy 1st reversed the pro-fibrotic phenotypes of fibroblasts

**FIGURE 3:**
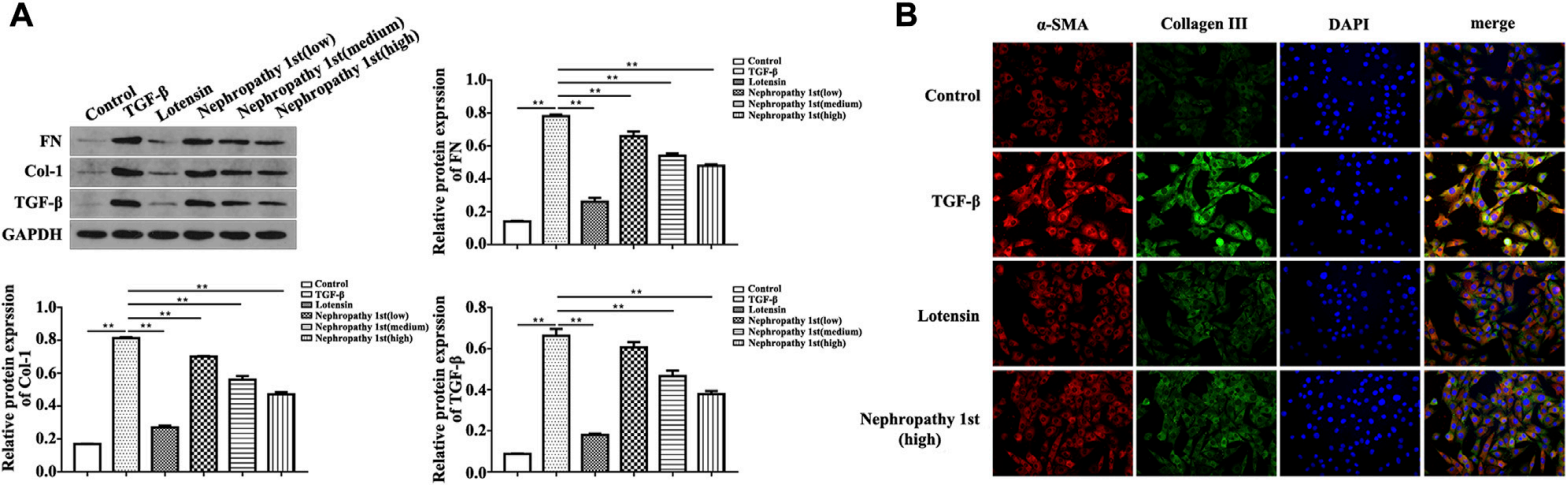
Nephropathy 1st inhibited the pro-fibrotic phenotypes in NRK-49F cells. Western blot was used to detect the expression of the biomarkers of renal fibrosis, including FN, col-1, and quantification of the expression of FN and col-1. changed to: Nephropathy 1st inhibited the pro-fibrotic phenotypes in NRK-49F cells. **(A)** Western blot was used to detect the expression of the biomarkers of renal fibrosis, including FN, col-1, and quantification of the expression of FN and col-1. **(B)** Immunofluorescence staining was used to detect the expression of α-SMA and collagen III. Data were representative of three independent experiments. *p < 0.05 and **p < 0.01.

After confirming the anti-fibrosis effect of Nephropathy 1st *in vivo*, we subsequently utilized TGF-β1 to induce rat fibroblast cells into myofibroblasts to evaluate the anti-fibrosis role Nephropathy 1st *in vitro*. TGF-β1-induced NRK-49F cells (a rat normal kidney fibroblast cell line) were treated with different doses of Nephropathy 1st for 72 h. A Western blot was used to detect the expression of renal fibrosis biomarkers including FN and col-1. TGF-β1 treatment promoted the expression of FN and col-1. Lotensin and the three concentrations of Nephropathy 1st significantly reversed TGF-β1 effects on Fn and col-1 expression. (Figure 3A). Immunofluorescence staining also showed that TGF-β1 treatment promoted the expression of α-SMA and collagen III, which was abrogated by lotensin or Nephropathy 1st treatment (Figure 3B).

### Nephropathy 1st attenuated rat renal fibrosis by activating the PPARγ signaling pathway

**FIGURE 4:**
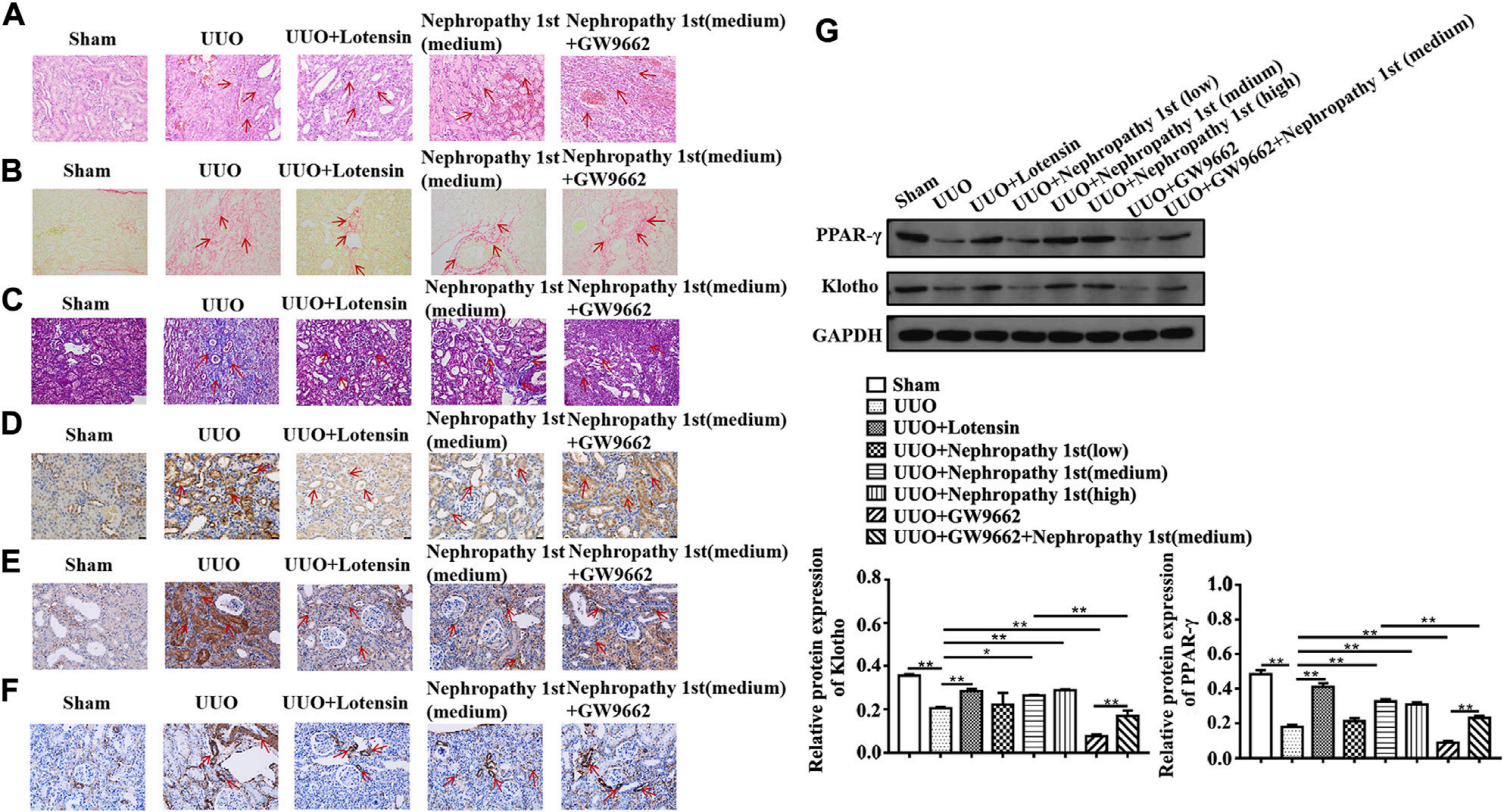
Nephropathy 1st attenuated rat renal fibrosis by activating the PPARγ/Klotho pathway *in vivo*. **(A)** HE staining, (B) Sirius Red Staining, **(C)** Masson staining, **(D–F)** TGF-β1, collagen III, and α-SMA IHC staining of different kidney tissues. **(G)** Expression of PPARγ and Klotho in the UUO group and lotensin group. Nephropathy 1st group and GW9662 (PPARγ inhibitor) group were analyzed by Western blot. ImageJ software was utilized for the measurement of the blot intensity of **(G)**. Data were representative of three independent experiments. *p < 0.05, **p < 0.01, and *p < 0.01 vs. control; ##p < 0.01 vs. model.

To further examine whether Nephropathy 1st attenuated renal fibrosis through the PPARγ pathway, GW9662, a PPARγ inhibitor, was used to treat fibrotic rats. HE staining indicated that although medium and high doses of Nephropathy 1st could relieve the degree of renal fibrosis, the therapeutic effect was abrogated by GW9662 treatment, as evidenced by the results that rats from the GW9662 group showed severe inflammatory cell infiltration and high expression of collagen III and α-SMA even after Nephropathy 1st administration. A Western blot also indicated that GW9662 markedly suppressed PPARγ and klotho expression in rat renal tissues (Figure 4).

**FIGURE 5:**
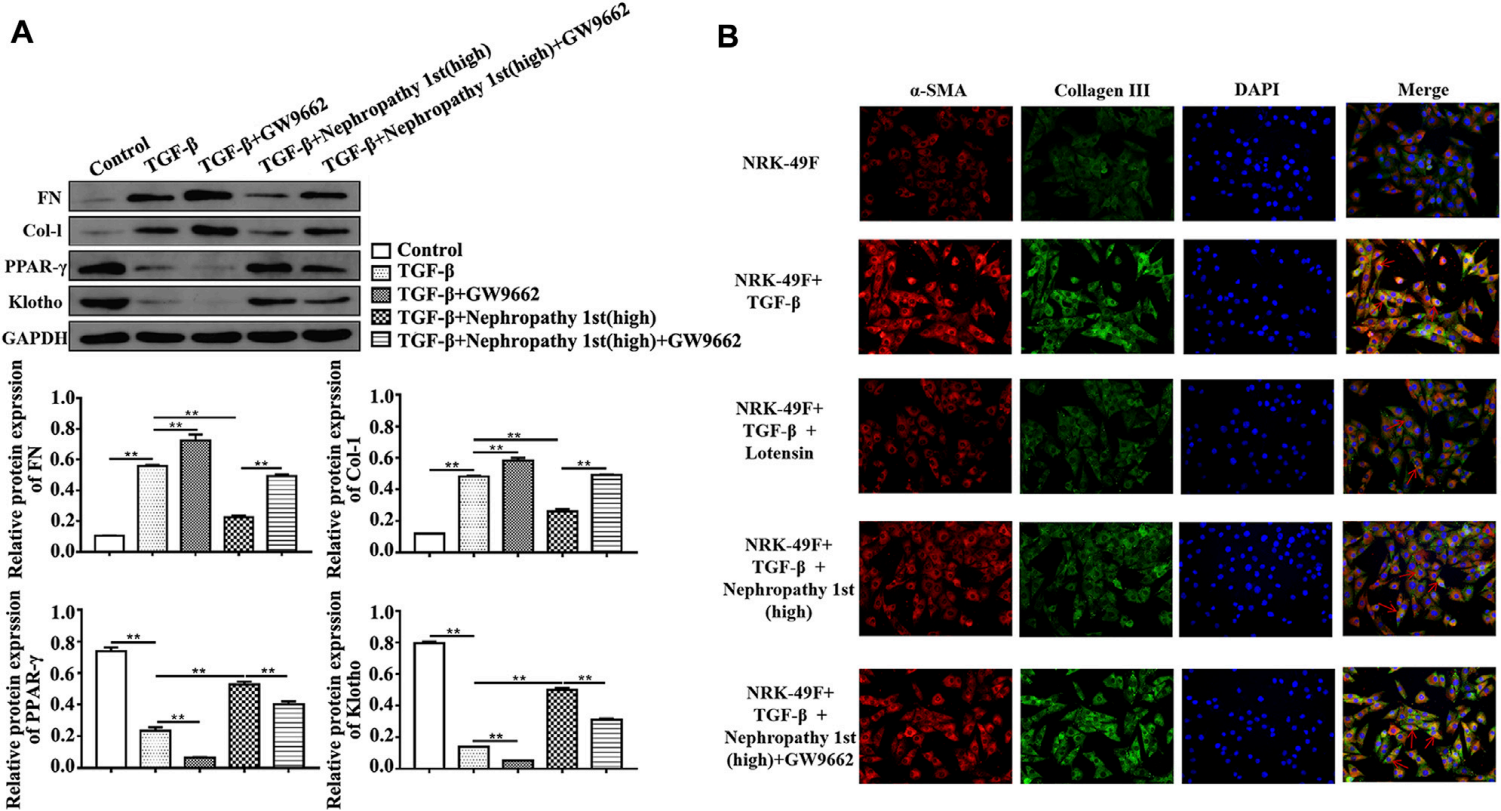
Inhibition of PPARγ by GW9662 abrogated the anti-fibrotic role of Nephropathy 1st. **(A)** Western blot showed the electrophoretic band of FN, Col-I, PPARγ, and Klotho; and the relative protein expression of FN, Col-I, PPARγ, and Klotho based on the gray value of WB. **(B)** Immunofluorescence (IF) technology showed the expression of α-SMA and collagen III protein level and the position in NRK-49F cells treated with TGF-β1 and GW9662 in the presence of lotensin or Nephropathy 1st. Data were representative of three independent experiments. *p < 0.05 and **p < 0.01.

In NRK-49F cells, we also found that lotensin and Nephropathy 1st reversed the effects of TGF-β1 on the expression of Col-I, FN, PPARγ, and Klotho. Importantly, GW9662 reversed Col-I and FN expression in Nephropathy 1st-treated fibroblasts (Figure 5). The aforementioned data indicated that Nephropathy 1st can alleviate renal fibrosis in rats by promoting PPARγ signaling activation.

### Metabolic analysis of serum metabolites: possible metabolic mechanism of nephropathy 1st

**FIGURE 6:**
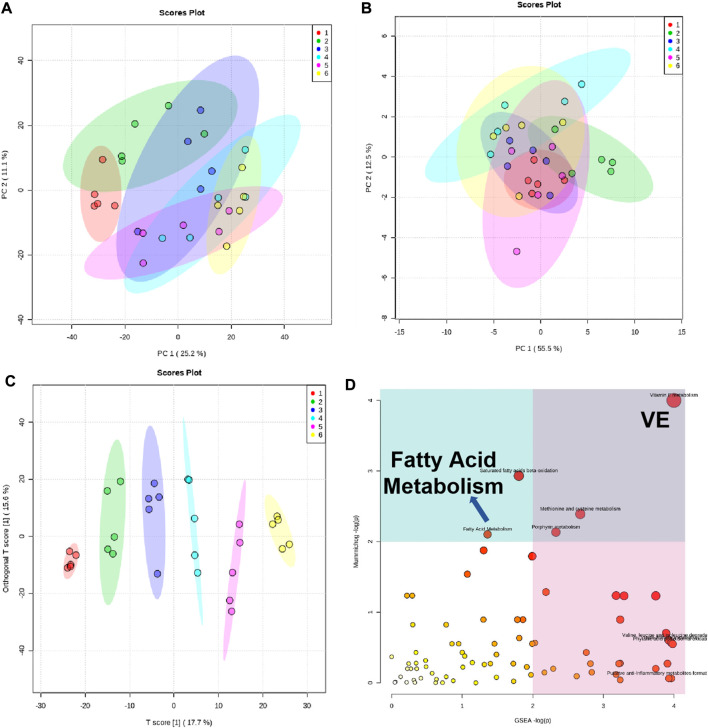
Analysis of metabolic content in the rat serum of groups 1–6. **(A)** PCA of the biological pathway according to the metabolic differences. Each point represented a component. **(B–C)**: PCA of the distribution of the 25 functional components we screened. **(D)** Analysis of the biological pathway according to the metabolic differences. Pathways associated with PPARγ (VE-related metabolism and fatty acid metabolism) are indicated.

Previous studies have shown that traditional Chinese medicine may exert biological effects by changing metabolic processes (Li T. et al., 2016; Deng H. F. et al., 2020a; Deng J. S. et al., 2020b; Liu et al., 2020). Therefore, metabonomic analysis was performed to measure the levels of serum metabolites in different groups of rats. In order to reduce the deviation, we homogenized the system. The metabolites with significant differences are listed in Supplementary Table S1. We first carried out principal component analysis (PCA) and found that compared with the sham group, the UUO group exhibited an upward trend in the PC2 direction (Figure 6A), which was reduced by lotensin treatment or Nephropathy 1st treatment in a similar manner, indicating that Nephropathy 1st may have a similar effect on rat renal fibrosis in terms of metabolic regulation. We next screened 25 possible functional components and analyzed the samples by PCA (Figures 6B,C). The results showed that the UUO group moved up diagonally which was reversed by the medium or high dose of Nephropathy 1st.

In order to further understand the pharmacological mechanism of Nephropathy 1st, we analyzed the biological pathways according to the metabolic differences, we used mummichog and gene set enrichment analysis (GSEA) algorithms, respectively, for biological pathway enrichment based on the KEGG database (Figure 6D). We only analyzed the biological pathways corresponding to the different spectrum peaks between the sham group with the UUO group, UUO group and the lotensin treatment group, and UUO group with the middle and high Nephropathy 1st treatment group. The results were enriched for some significantly changed metabolites, as shown in Figure 6D. In particular, we noticed changes in VE-related metabolism and fatty acid metabolism, and the pathways of these two metabolites were closely related to PPARγ. This also confirms that the effect of Nephropathy 1st is achieved by affecting the PPARγ pathway, which is consistent with the reduced PPARγ expression by Nephropathy 1st. The results suggest that Nephropathy 1st may improve renal fibrosis by altering the production of certain types of metabolites and the related PPARγ signaling pathways.

## Discussion

In this study, Nephropathy 1st was found to suppress renal fibrosis *in vivo* and *in vitro*. Mechanistically, Nephropathy 1st regulated fatty acid metabolism *via* activating PPARγ signaling pathways, the inactivation of which contributed to renal fibrosis.

Recently, increasing evidence has focused on the use of traditional Chinese medicine (TCM) in the treatment of renal diseases. For instance, Huaiqihuang suppressed kidney damage *via* regulating energy metabolism, oxidative response, and immune function (Li T. et al., 2016). *Cordyceps cicadae* mycelia inhibited cisplatin kidney injury *via* alleviating oxidative stress and inflammatory responses (Deng JS. et al., 2020). Bu-Shen-Jiang-Ya decoction suppresses hypertensive renal damage and renal fibrosis *via* targeting TGF-β/SMAD signaling (Liu et al., 2020). Aristolactam I suppressed the ferroptosis of renal tubular epithelial cells *via* activating Nrf2-HO-1/GPX4 signaling (Deng HF. et al., 2020). These results suggested that TCM may have anti-inflammation, anti-oxidant, and anti-fibrosis properties in the treatment of nephropathy. In this study, Nephropathy 1st suppressed renal fibrosis, as manifested by the decreased expression of α-SMA and collagen III in Nephropathy 1st-treated rats, and reduced the pro-fibrotic phenotypes of fibroblasts. The major ingredients in Nephropathy 1st include Radix Bupleuri, *Scutellaria baicalensis* Georgi, Ginger *Pinellia tuberifera*, *Paeonia lactiflora* Pall, China root, *Scutellaria barbata*, *Smilax china*, and *Stemmacanthauniflora* Dittrich. It is reported that Radix Bupleuri ameliorates LPS-induced acute lung injury in mice (Du et al., 2018) and attenuates renal fibrosis by the Hedgehog pathway (Ren et al., 2020). *Scutellaria baicalensis* Georgi is a major bioactive compound and has multiple biological activities, including anti-inflammatory, antitumor, and antibacterial effects (Liao et al., 2021). These findings suggest that Nephropathy 1st may be endowed with anti-inflammatory and anti-fibrotic properties, which is consistent with this study.

PPARγ is a ligand-dependent transcription factor belonging to the nuclear hormone receptor family (Marion-Letellier et al., 2016) and is highly expressed in renal tissues. However, the dysfunctioned PPARγ is closely associated with the pathogenesis of nephropathy. For instance, PPARγ was downregulated in diabetic nephropathy (Zhang and Guan 2005). Additionally, downregulated PPARγ expression may induce ischemia in reperfusion-induced acute kidney injury (Singh et al., 2019). In contrast, overexpression of PPARγ maintained glomerulosclerosis and alleviated renal injury (Matsushita et al., 2016). In this study, PPARγ was also found to be downregulated in renal fibrosis models, while Nephropathy 1st treatment activated PPARγ signaling. However, the inhibition of PPARγ abrogated the beneficial effects of Nephropathy 1st and promoted renal fibrosis *in vivo* and *in vitro*. These results suggested that Nephropathy 1st inhibits renal fibrosis *via* activating PPARγ signaling. In our report, we revealed that the therapeutic role of Nephropathy 1st was dependent on the activation of PPARγ signaling.

PPARγ is a central modulator of fatty acid oxidation (FAO), which is the preferred energy source for highly metabolic cells. Dysregulated FAO is complicated as an effector pathway in the pathophysiology of nephropathy (Kang et al., 2015). FAO dysfunction contributes to cisplatin-induced acute kidney injury (AKI) (Li M. et al., 2020). Overexpressed fatty acid-binding protein 4 promotes the apoptosis of human mesangial cells (HMCs) and exacerbates diabetic nephropathy (Yao et al., 2015). As a key regulator of FAO, PPARs mediate fatty acid metabolism and alleviate nephropathy. For instance, the activation of AMPK/PPARα inhibits podocyte injury (Wu et al., 2021). RB394, a new PPARγ agonist, suppresses the renal interstitial fibrosis (Stavniichuk et al., 2020). Therefore, activation of the FAO signaling pathway may be a promising strategy for the treatment of renal fibrosis.

## Conclusion

In conclusion, Nephropathy 1st suppressed renal fibrosis *via* activating PPARγ signaling. Hence, Nephropathy 1st may serve as a potential strategy for renal fibrosis.

## Data Availability

The datasets presented in this study can be found in online repositories. The names of the repository/repositories and accession number(s) can be found at: www.ncbi.nlm.nih.gov; PRJNA868522.
